# High waist-to-height ratio and associated factors in adolescents from a city in Southern Brazil: a cross-sectional study

**DOI:** 10.1590/1984-0462/2022/40/2020468

**Published:** 2021-10-04

**Authors:** Mateus Augusto Bim, André de Araújo Pinto, Gaia Salvador Claumann, Andreia Pelegrini

**Affiliations:** a Universidade do Estado de Santa Catarina, Florianópolis, SC, Brazil.

**Keywords:** Body composition, Adolescent, Obesity, abdominal, Composição corporal, Adolescente, Obesidade abdominal

## Abstract

**Objective:**

To verify the prevalence of abdominal obesity with the waist-to-height ratio (WHtR) and associated factors in adolescents from a city in Southern Brazil.

**Methods:**

A total of 960 adolescents (494 boys) aged 15–18 years old participated in this study. The dependent variable was WHtR; independent variables were self-reported age, economic level, sexual maturation, physical activity level, screen time, and body fat. Data were analyzed using descriptive statistics and logistic regression.

**Results:**

It was observed that 36.7% of the adolescents presented high WHtR (50.2% in girls and 23.9% in boys). Regardless of sex, adolescents with high body fat were more likely of having high WHtR (boys: *Odds Ratio* [OR] 29.79; 95% confidence interval [95%CI] 16.87–52.62; girls: OR 19.43; 95%CI 10.51–35.94). In girls, high WHtR was associated with age (OR 1.83; 95%CI 1.17–2.87), and in boys, with economic level (OR 2.34; 95%CI 1.01–5.45).

**Conclusions:**

One in each three adolescents has abdominal obesity. Among adolescents with high body fat, girls aged 15–16 and boys with high-income are the groups most exposed to abdominal obesity.

## RESUMO

**Objetivo:**

Verificar a prevalência de obesidade abdominal, por meio da razão cintura-estatura (RCE), e fatores associados em adolescentes de uma cidade do sul do Brasil.

**Métodos:**

Participaram 965 adolescentes (499 meninos) de 15 a 18 anos. A variável dependente foi a RCE elevada e as variáveis independentes foram idade, nível econômico, maturação sexual, nível de atividade física, tempo de tela e adiposidade corporal. Os dados foram analisados por meio de estatística descritiva e regressão logística.

**Resultados:**

36,7% dos adolescentes apresentaram RCE elevada (50,2% das meninas e 23,9% dos meninos). Independentemente do sexo, os adolescentes com adiposidade corporal elevada apresentaram maiores chances de terem RCE elevada (masculino: OR 30,91; IC95% 17,37–55,00; feminino: OR 19,18; IC95% 10,39–35,40). Nas meninas, observou-se associação da RCE elevada com idade (OR 1,88; IC95% 1,20–2,94) e, nos meninos, com nível econômico (OR 2,35; IC95% 1,01–5,46).

**Conclusões:**

Um em cada três adolescentes tem obesidade abdominal. Adolescentes com adiposidade corporal elevada, meninas de 15–16 anos e meninos de nível econômico alto são os grupos mais expostos à obesidade abdominal.

## INTRODUCTION

Obesity, considered a serious public health problem, which was previously observed only in high-income countries, has been a growing concern in low- and middle-income countries.^1^ From the point of view of its body distribution, abdominal obesity, determined by excessive fat accumulation in the abdominal region, presents greater health risks when compared to general obesity.^2^ Fat concentrations in the abdomen region increase the risk of developing nonalcoholic fatty liver disease, liver cirrhosis, type 2 diabetes mellitus, and cardiovascular diseases,^3^ and is directly associated with metabolic syndrome.^2^

Studies that investigated abdominal obesity in adolescents have been developed worldwide to observe the prevalence and provide control of this epidemic.^4^ ^–^ ^6^ In this context, anthropometric indicators are often used to estimate body composition due to their practicality in measurements, especially in large samples, and the most widely used in estimating abdominal obesity are waist circumference (WC) and waist-to-height ratio (WHtR).^4^ ^–^ ^6^ WHtR seems to be an indicator of better accuracy for the estimation of abdominal obesity due to its close relationship with metabolic syndrome when compared to WC.^7^

In addition to prevalence, understanding the factors associated with abdominal obesity is important. In this context, few studies have investigated the factors associated with high WHtR in the adolescent population. The findings of these studies indicate an association of high WHtR with high economic level,^8^ and with less satisfaction towards self-perceived appearance.^9^

Considering the health risks related to abdominal obesity, knowing its context and associated factors can help in the planning of prevention and health promotion strategies, avoiding major complications. Given the above, the present study aimed to verify the prevalence of abdominal obesity and its association with age, economic level, sexual maturation, body fat, physical activity level, and sedentary behavior based on screen time in Brazilian adolescents.

## METHOD

This study is characterized as epidemiological, school-based with cross-sectional design, approved by the institutional ethics committee under opinion No. 2.172.699/2017.

Study population consisted of adolescents enrolled in public high schools of Florianópolis City, Santa Catarina state, Southern Brazil. For sample selection, stratification was performed by regions from Florianópolis City, adopting the distribution criteria according to the Municipal Health Secretariat (Northern, Southern, Eastern, Downtown, and Continent regions). For sample calculation, recommendations suggested by Luiz and Magnanini^10^ were followed. The confidence level used was 1.96, design effect of 1.5, tolerable error of 4%, and prevalence estimation of 50% (unknown outcome), with 10% increase for possible refusals and loss or incorrect questionnaire completion. Considering the target population of 10,192 adolescents enrolled in 2017 (data from the State Secretariat of Education), a minimum sample of 936 individuals was estimated. The largest school in each region was selected to collect the data during the second half of 2017 and the first half of 2018.

After selecting the schools, the number of classes needed to reach the estimated number of adolescents determined for each region (saturation sampling) was drawn. All students in the selected classes were invited to participate in research (cluster sampling). All adolescents aged 15 to 18 years old (both sexes), enrolled in selected schools, who did not have physical limitations that prevented them from participating in physical tests, and who presented the Free and Informed Consent Term signed by a guardian and the consent form signed by the adolescent themselves were included.

High WHtR (dependent variable) was calculated by the relationship between waist circumference^11^ and height,^11^ both measurements in centimeters. The high WHtR was classified with the cutoff points by Pelegrini et al.^12^ for adolescents aged 15–17 years old (boys: ≥0.43; girls: ≥0.41) and for 18-year-old adolescents, the cutoff point by Ashwell and Hsieh^13^ (≥0.50) was used.

Age was categorized into 15–16 and 17–18. Economic level was identified by the Brazilian Economic Classification Criterion,^14^ in which individuals were categorized as high (A+B1+B2) and medium/low (C1+C2+D+E) economic level. Sexual maturation was assessed using the figures that refer to the development of pubic hair, developed by Adami and Vasconcelos,^15^ based on the figures of Tanner,^16^ in which adolescents indicated the stage corresponding to their current pubic hair development. Adolescents were classified as prepubertal, pubertal, and postpubertal; however, as there was no adolescent in the prepubertal stage, only the pubertal and postpubertal categories were used in the analyzes.

Body fat was estimated by the sum of the triceps and subscapular skinfolds, which were measured twice non-consecutively in the right body side,^11^ using a scientific Cescorf^®^ adipometer (0.1mm resolution). To determine body fat, the values for triceps and subscapular skinfold measurements were summed and classified according to sex.^17^ Due to the low frequency of adolescents in the low body fat category, for statistical analysis, it was categorized into normal (low+normal body fat) and high (high body fat) body fat.

Screen time (television, computer, and video games) was measured in how many hours they remained in front of these electronic devices (separately) on a usual day during the week (Monday to Friday) and on weekends (Saturday and Sunday). To calculate the weekly time using each device, the following equation was used: [(weekday time×5+weekend time×2)/7]. Total screen time was determined by the sum of total TV, computer, and video game time.^18^ The cutoff point for excessive screen time was four hours or more.^6^

Physical activity level was assessed by the International Physical Activity Questionnaire (IPAQ — short version),^19^ regarding the practice of physical activity of adolescents in the last seven days. The cut-off point of 60 minutes of moderate-to-vigorous physical activity was used to classify adolescents aged 15 to 17 years old, and 150 minutes per week for adolescents aged 18 years old.^20^

The study included adolescents aged 15–18, enrolled in schools selected to participate in the study, who were present at school on the day of data collection, without physical limitations that prevented them from participating in the tests, and having provided signed consent and assent forms.

Statistical procedures were performed using the IBM *Statistical Package for the Social Sciences* (SPSS) 20.0 software, using descriptive statistics. Kolmogorov-Smirnov test was used to verify data distribution. After log_10_ transformation, the variables video game time and total screen time began to show distribution normality. Differences in the mean values of variables between sexes were analyzed by the Mann-Whitney U test and Student's t-test for independent samples. Chi-square was used to verify differences in the proportions of variables between sexes; for associations, binary logistic regression was used by the crude and adjusted analysis, estimating *odds ratio* and 95% confidence intervals (95%CI). The significance level adopted was p<0.05.

## RESULTS

Study sample consisted of 960 adolescents (51.5% boys) aged 15–18 years old. The prevalence of abdominal obesity was 36.7% (boys: 23.9%; girls: 50.2%). All variables showed differences in mean values between sexes, except for WHtR. The means of triceps and subscapular skinfolds, sum of skinfolds, and television time were higher in girls; the means of the other variables were higher in boys (Table 1).

**Table 1:** Sample characterization represented as mean and standard deviation.

	Total (n=960)	Male (n=494)	Female (n=466)	p-value
	Mean (SD)	Mean (SD)	Mean (SD)	
Age (Years old)†	16.46 (0.95)	16.56 (0.97)	16.36 (0.93)	<0.001
Height (cm)‡	171.60 (8.77)	177.20 (7.02)	165.68 (6.16)	0.002
Body mass (kg)†	62.51 (12.99)	66.72 (12.93)	58.05 (11.49)	<0.001
WC (cm)†	71.63 (8.67)	73.49 (8.43)	69.66 (8.50)	<0.001
∑2SF (mm)†	26.72 (13.18)	21.77 (11.78)	31.97 (12.55)	<0.001
WHtR (cm)†	0.42 (0.05)	0.42 (0.05)	0.42 (0.05)	0.050
Television (min/day)†	119.77 (135.41)	110.67 (136.72)	129.41 (133.47)	<0.001
Computer (min/day)†	119.42 (165.23)	148.87 (178.42)	88.20 (143.71)	<0.001
Videogame (min/day)‡	67.26 (133.91)	109.05 (160.07)	22.97 (77.43)	<0.001
Total screen time (min/day)‡	306.45 (278.17)	368.58 (306.88)	240.58 (226.46)	0.001
MVPA (min/week)†	96.83 (141.18)	600.18 (735.90)	345.75 (557.30)	<0.001

SD: standard deviation; WC: waist circumference; ∑2SF: sum of two skinfolds; WHtR: waist-to-height ratio; MVPA: moderate/vigorous physical activity; kg: kilograms; cm: centimeters; mm: millimeters; min: minutes;

† Mann-Whitney U test;

‡ independent Student's t-test.

Table 2 shows associations between elevated WHtR and independent variables in boys. In the crude analysis, adolescents with high economic level (OR 2.19; 95%CI 1.12–4.29), at the post-pubertal sexual maturation stage (OR 1.67; 95%CI 1.08–2.58), and with high body fat (OR 27.93; 95%CI 16.27–47.97) were more likely to present high WHtR. After adjustment, economic level and body fat remained associated with the outcome, revealing that adolescents with high economic level (OR 2.34; 95%CI 1.01–5.45) and those with high body fat (OR 29.79; 95%CI 16.87–52.62) were more likely to present high WHtR compared to their peers.

**Table 2:** Association between abdominal obesity and independent variables in male adolescents.

		n (%)	High WHtR			
			Crude analysis	p-value	Adjusted analysis	p-value
			OR (95%CI)		OR (95%CI)	
Age						
	15–16 years old	55 (46.60)	1	0.676	1	0.143
	17–18 years old	63 (53.40)	0.92 (0.60–1.39)		0.66 (0.37–1.15)	
Economic level						
	High	107 (90.70)	2.19 (1.12–4.29)	0.023	2.34 (1.01–5.45)	0.048
	Medium/low	11 (9.30)	1		1	
Sexual Maturation						
	Pubertal	39 (33.10)	1	0.020	1	0.367
	Postpubertal	79 (66.90)	1.67 (1.08–2.58)		1.30 (0.74–2.27)	
Body fat						
	Normal	32 (27.10)	1	<0.001	1	<0.001
	High	86 (72.90)	27.93 (16.27–47.97)		29.79 (16.87–52.62)	
Physical activity						
	Meets recommendations	53 (44.90)	1	0.117	1	0.864
	Does not meet recommendations	65 (55.10)	1.39 (0.92–2.11)		1.05 (0.60–1.82)	
Screen time						
	<4h	42 (35.60)	1	0.299	1	0.566
	≥4h	76 (64.40)	1.26 (0.82–1.93)		1.18 (0.67–2.09)	

WHtR: waist-to-height ratio; OR: *Odds Ratio*; 95%CI: 95% confidence interval.

In girls, crude analysis showed that adolescents with high body fat (OR 17.55; 95%CI 9.65–31.93) and those who did not meet physical activity recommendations (OR 1.57; 95%CI 1.07–2.30) were more likely of presenting abdominal obesity. After adjustment, adolescents aged 15 and 16 years old (OR 1.83; 95%CI 1.17–2.87) and with high body fat (OR 19.43; 95%CI 10.51–35.94) were more likely of presenting abdominal obesity (Table 3).

**Table 3:** Association between abdominal obesity and independent variables in female adolescents.

		n (%)	High WHtR			
			Crude analysis	p-value	Adjusted analysis	p-value
			OR (95%CI)		OR (95%CI)	
Age						
	15–16 years old	130 (55.60)	1.32 (0.91–1.89)	0.139	1.83 (1.17–2.87)	0.008
	17–18 years old	104 (44.40)	1		1	
Economic level						
	High	190 (81.20)	1.04 (0.66–1.65)	0.871	1.25 (0.71–2.19)	0.434
	Medium/low	44 (18.80)	1		1	
Sexual maturation						
	Pubertal	157 (67.10)	1.05 (0.72–1.55)	0.793	0.96 (0.60–1.53)	0.864
	Postpubertal	77 (32.90)	1		1	
Body fat						
	Normal	110 (47.0)	1	<0.001	1	<0.001
	High	124 (53.0)	17.55 (9.65–31.93)		19.43 (10.51–35.94)	
Physical activity						
	Meets recommendations	56 (23.90)	1	0.027	1	0.213
	Does not meet recommendations	178 (76.10)	1.58 (1.05–2.37)		1.35 (0.84–2.18)	
Screen time						
	<4h	142 (60.70)	1	0.865	1	0.446
	≥4h	92 (39.30)	0.97 (0.67–1.40)		0.84 (0.54–1.31)	

WHtR: waist-to-height ratio; OR: *Odds Ratio*; 95%CI: 95% confidence interval.

## DISCUSSION

The prevalence of abdominal obesity in adolescents in Florianópolis city was 36.7%. In Spanish adolescents aged 12 to 17, the prevalence was 14.3%, with 20.0% for boys and 8.7% for girls.^8^ Similar results to those of present study were found in adolescents from New Caledonia, an archipelago located in Oceania, in which 32.4% had high WHtR, 28.8% of boys, and 35.7% of girls.^9^ In Brazil, a study carried out in Viçosa City, Minas Gerais state, with adolescents aged 14 to 19, observed a prevalence of abdominal obesity in 11.6% of the sample.^4^ In Rio Grande do Sul State, the prevalence of abdominal obesity as measured by the WHtR was 18.3%.^5^

It was observed that the prevalence of abdominal obesity varies by region, with the highest prevalence being found in cities in Southern Brazil, which may be related to different environmental and cultural aspects in each region of the country. When comparing to other Brazilian and international studies, the prevalence of abdominal obesity in the investigated sample is high, which is worrying, since obesity tends to remain until adulthood,^21^ being also related to the development of metabolic syndrome.^7^

Another point to be highlighted is the higher prevalence of abdominal obesity in girls, different from most other evidence, that indicates a higher prevalence in boys.^8^ A possible justification for such findings may be related to hormonal and/or behavioral aspects, since the proportion of girls who do not meet the recommendations for physical activity was higher than that of boys (71.5 vs. 48.8%; p<0.001 — data not shown). However, according to a systematic review that investigated the prevalence of abdominal obesity (measured by waist circumference) in adolescents, it was not possible to identify consensus regarding differences by sex.^22^ Thus, inconsistency of prevalence may also be related to several factors, for example, unhealthy lifestyle behaviors,^23^ place of residence, and culture.^24^

In the present study, positive associations were observed between high WHtR and body fat in both sexes. Even though they are indicators of different body fat distributions (peripheral and central), higher body fat levels are believed to be related to higher waist circumference measurements due to the collinearity that seems to exist among body fat measurements (peripheral, central, and total).^25^

Higher-income boys are more exposed to abdominal obesity. In a study that followed Chinese individuals for 18 years, those with higher economic status were more likely of presenting abdominal obesity.^26^ Adolescents with higher economic level enjoy goods and present behaviors that may be associated with abdominal obesity, such as technologies that keep them in sedentary activities,^6^ non-active commuting means,^27^ and poor nutritional-quality foods.^28^

There was no association between elevated WHtR and sexual maturation. However, the association of the outcome with age, in girls, may be related to changes in the levels of sexual and growth hormones that occur in the puberty phase, reflecting changes in the body weight and fat distribution.^29^ All the girls in the present study were at least in their pubertal phase, and possibly most of them are in the youngest age group (15-16 years old), therefore, they are the ones who are going through more intense maturation changes, which may explain the greater values of the waist-to-height ratio. Moreover, older adolescents may be more concerned with maintaining a slim body, possibly due to greater knowledge of the harms of high body adiposity and to suffering pressures and influences from peers, family members, and the media about having a body that meets their needs and current social standards. This could explain, in part, the greater chance of younger adolescents having elevated WHtR.

This study has some limitations, such as the memory bias of those evaluated in relation to screen time and physical activity, as well as the population referring only to public school students, which may not reflect the same reality as students from private schools may have. However, to the best of our knowledge, this is one of the first studies investigating factors associated with abdominal obesity using the WHtR indicator in Brazilian adolescents.

In conclusion, the results indicated that one in every three adolescents has high WHtR. Adolescents of both sexes with higher body fat and girls aged 15–16 years old are more likely to present high WHtR. Further studies to investigate the prevalence of abdominal obesity by WHtR and associated factors in different educational contexts and regions of Brazil are needed. Such evidence can serve as a basis for the formulation of health education strategies in schools, which should be prioritized for Brazilian public health, aiming at the prevention of abdominal obesity.
